# Quantifying Hand Strength and Isometric Pinch Individuation Using a Flexible Pressure Sensor Grid

**DOI:** 10.3390/s23135924

**Published:** 2023-06-26

**Authors:** Brian J. Conway, Léon Taquet, Timothy F. Boerger, Sarah C. Young, Kate B. Krucoff, Brian D. Schmit, Max O. Krucoff

**Affiliations:** 1 Medical College of Wisconsin, Milwaukee, WI 53226, USA; 2 Department of Neurosurgery, Medical College of Wisconsin, Milwaukee, WI 53226, USAmaxkrucoff@gmail.com (M.O.K.); 3 Department of Plastic & Reconstructive Surgery, Medical College of Wisconsin, Milwaukee, WI 53226, USA; 4 Department of Biomedical Engineering, Marquette University and Medical College of Wisconsin, Milwaukee, WI 53226, USA

**Keywords:** finger isometric individuation, hand strength, hand motor function, Tekscan, neurophysiology

## Abstract

Modulating force between the thumb and another digit, or isometric pinch individuation, is critical for daily tasks and can be impaired due to central or peripheral nervous system injury. Because surgical and rehabilitative efforts often focus on regaining this dexterous ability, we need to be able to consistently quantify pinch individuation across time and facilities. Currently, a standardized metric for such an assessment does not exist. Therefore, we tested whether we could use a commercially available flexible pressure sensor grid (Tekscan F-Socket [Tekscan Inc., Norwood, MA, USA]) to repeatedly measure isometric pinch individuation and maximum voluntary contraction (MVC) in twenty right-handed healthy volunteers at two visits. We developed a novel equation informed by the prior literature to calculate isometric individuation scores that quantified percentage of force on the grid generated by the indicated digit. MVC intra-class correlation coefficients (ICCs) for the left and right hands were 0.86 (*p* < 0.0001) and 0.88 (*p* < 0.0001), respectively, suggesting MVC measurements were consistent over time. However, individuation score ICCs, were poorer (left index ICC 0.41, *p* = 0.28; right index ICC −0.02, *p* = 0.51), indicating that this protocol did not provide a sufficiently repeatable individuation assessment. These data support the need to develop novel platforms specifically for repeatable and objective isometric hand dexterity assessments.

## 1. Introduction

Hand function is one of the most complex components of the human motor system [1,2,3,4,5]. Grasping objects, for example, often begins with visual perception and generation of a motive to reach [6,7,8]. Motor planning in the frontal and parietal cortices is then followed by upper motor neurons in the primary motor cortex (M1) firing to activate their distal counterparts in the spinal cord [9]. This signal is then translated across the neuromuscular junction to the muscles of the hand and the upper extremity in motor units [10,11]. Successful completion of a task, such as picking up a cup to drink, is then complemented by a variety of sensory neurons activating upon contact with the object and a resultant modulation of applied force through the thalamus, primary sensory cortex (S1), and cerebellum, amongst other integrative sites [12,13]. While the entire sequence may occur in a matter of seconds, the multiple neurological and musculoskeletal resources essential to this process means that it can be disrupted by a multitude of central or peripheral injuries [14,15,16,17]. Damage to any part of the hand motor system from the brain and spinal cord to peripheral nerves and intrinsic muscles of the hand can result in unique and sometimes subtle deficits, all of which can impact daily functioning and quality of life [3,18,19,20].

Clinically, subjective assessments of hand grip strength are often translated into “manual muscle testing (MMT)”, a scoring system ranging from 0 to 5, where 0 represents no muscle firing and 5 represents no perceived neurological deficit (i.e., full strength for that participant) [21]. Although this assessment is intended to be objective, the score can vary drastically based on the examiners’ perception and resistance provided, and it does not include any assessments of dexterity or motor control [22]. Many research labs and clinics use digital dynamometers to quantify the total force exerted by the hand during a trial of maximum voluntary contraction (MVC) as an objective adjunct to MMT for hand strength [23,24]. However, this technique is a gross assessment that does not detect subtle deficits in hand function [25,26,27]. Moreover, there is no commercially available digital dynamometer designed to measure dexterous modulation of individual finger strength, which is a core component of tasks required for daily living [1,2,28,29,30,31,32,33].

To better quantify assessments of hand dexterity, we and others have previously developed techniques to score kinematic finger individuation, or the ability to flex a single digit in isolation [1,2,29,34,35,36,37,38,39,40]. Since isometric kinetic components of force generation are also critical but involve slightly different neural pathways [41,42,43], others have applied similar techniques to study isometric finger individuation [1,2,29,34]. Quantification of isometric finger individuation is essential, as grasping and manipulating objects between the thumb and another digit is integral to everyday interactions with our environment [1,2,34,40].

For example, McCall et al. assessed isometric finger individuation in pediatric participants with cerebral palsy using forces obtained with five load cells [11]. Wolbrecht et al. designed a robotic device that can apply variable resistance to assess and rehabilitate finger strength and dexterity in individuals with corticospinal tract injury due to stroke [2]. Others have developed different intricate platforms to measure isometric individuation [3,19,33,37,44]. However, despite this foundational work, there is no generally accepted technique or commercially available device that has been assessed for repeatability and which can easily be used across centers. Additionally, there is no published literature on the repeatability of platforms measuring isometric pinch individuation, which is essential to interpreting results over time [1,2,45].

Therefore, here we assessed the repeatability of quantitative pinch individuation and MVC measurements obtained using a commercially available flexible pressure sensor grid (Tekscan F-Socket [Tekscan Inc., Norwood, MA, USA]). The aim of this study was to assess the repeatability of quantitative pinch individuation and MVC measurements obtained using this platform in a set of healthy, homogenous volunteers. If successful, such a simple platform might be used by clinical specialties of neurosurgery, orthopedics, plastic surgery, physical medicine and rehabilitation, and physical/occupational therapy to assess deficits and recovery of hand dexterity across time and location.

## 2. Materials and Methods

### 2.1. Ethical Approval of Subjects

Twenty adult participants were recruited (sixteen female and four male; all right-hand dominant) (Table 1). Inclusion criteria were as follows: age 18+, able to understand a written informed consent and willing to sign it, possessed normal hand strength (5/5 on MMT), and willing to participate in all aspects of the study. Exclusion criteria were as follows: had a history of malignancy in the last 3 years, possessed decreased hand motor strength (i.e., 4/5 or less), or had a hand deformity or injury that interfered with their ability to perform hand grip tasks. Written informed consent was obtained from all twenty participants. This study received approval from the Medical College of Wisconsin (MCW) Institutional Review Board (IRB) (PRO00040521) and the Froedtert Health Office of Clinical Research and Innovative Care Compliance (OCRICC).

### 2.2. Isometric Pinch and Grasp Tasks

**Figure 1:**
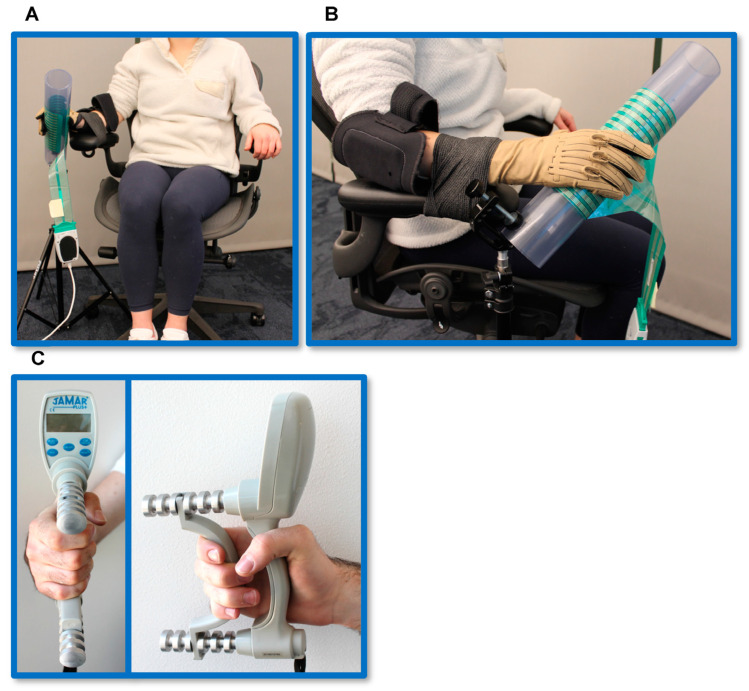
Experimental setup. (**A**,**B**) Participant in position for force trials with the Tekscan F-Socket while wearing Cyberglove III. The Tekscan F-Socket flexible pressure sensor grid is fastened to the PVC pipe and oriented at a 45° angle. The participant’s wrist is stabilized to the arm of the office chair with athletic adhesive bandage. (**C**) The JAMAR digital dynamometer was used in addition to the trials of whole-hand MVC.

**Figure 2:**
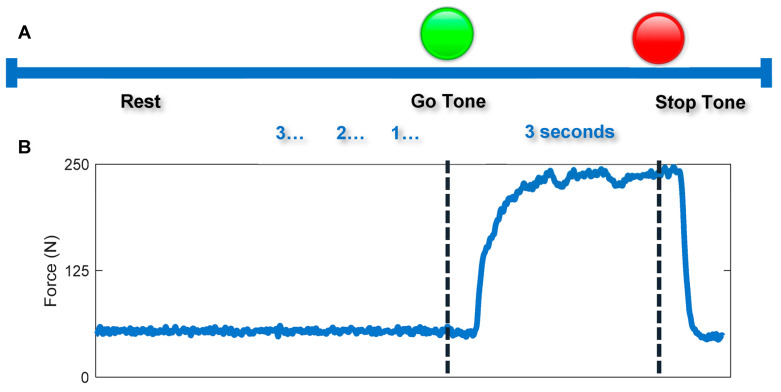
Isometric pinch and grasp strength task timeline of events. (**A**) Timeline of events during a trial beginning with a rest period of 30 s for isometric individuation and 1 min for MVC followed by a countdown, cue to exert force for three seconds, and a stop tone. (**B**) Tekscan data from a trial of MVC.

Data were collected from both hands in all the twenty participants at two study visits separated by at least two weeks. Participants sat in an office chair while wearing the Cyberglove III (CyberGlove Systems, San Jose, CA, USA), a commercially available data glove that tracks finger joint position to ensure all movements were isometric [29,40,46,47]. Participants’ forearms were fastened to the office chair in a wrist-neutral position using athletic adhesive bandage to control for extraneous movement as much as possible (Figure 1A,B). Their hand was rested on a commercially available flexible pressure grid (Tekscan F-Socket [Tekscan Inc., Norwood, MA, USA]) measuring 23.5 cm × 9 cm, consisting of 240 pressure sensors arranged in a 15 × 14 array, wrapped around a PVC pipe (7.5 cm). The PVC pipe was oriented at a 45-degree angle and attached to a tripod stand with a clamp to comfortably fit to each participant’s hand. Participants performed four trials of whole-hand MVC and isometric pinch grip individuation with each indicated digit opposed to the thumb on each hand. During pinch grip trials, the participant’s hand remained in contact with the mat, but they were instructed to only apply force with the thumb and indicated finger. They were also instructed to not elevate any other finger. The order in which hands were tested and strength tasks were administered was randomized, and during the first trial, participants were instructed to exert sub-maximum force to become accommodated with the motor task in accordance with the NIH Toolbox guidelines of measuring hand grip strength [48]. Sub-maximum trials were not included in data analysis. A visual prompt was provided with text indicating which finger(s) to squeeze followed by a countdown from three seconds. To indicate the start of a task, a light changed from red to green and a tone was played. All trials lasted three seconds with a one-minute rest between the trials of whole-hand MVC and 30 seconds between the trials of pinch grip individuation (Figure 2) [48]. During the following three trials of maximum grip-strength, participants were provided with words of encouragement by the two individuals collecting the data who repeatedly shouted “Go!” as words of encouragement are associated with enhanced performance [49,50,51]. Participants also performed one sub-maximum and one maximum trial of whole-hand MVC using the JAMAR digital dynamometer (Patterson Medical, Cedarburg, WI, USA) with a one-minute rest between the two trials (Figure 1C). The JAMAR digital dynamometer is a validated clinical device that measures maximum grip force in Newtons [24]. We used it as a comparator for our novel measurement technique.

### 2.3. Data Analysis

#### 2.3.1. Whole-Hand Grasp Strength

All data were processed and analyzed using MATLAB© (R2020a). Peak MVC force per individual per trial was calculated by finding the largest total force (i.e., sum of all sensors) applied to the mat during the strength task. Repeatability of these measurements was assessed by calculating intra-class correlation coefficients (ICC) of mean MVC for each hand and study visit. Violin and Bland–Altman plots were developed to visualize the data distribution [52]. Minimal detectable change (MDC) was also calculated according to the standards outlined by the Shirley Ryan Ability lab (1.96 × Standard error of the mean × square root of 2). To compare our platform to a validated clinically used digital dynamometer, [24] the first maximum effort MVC trial conducted using Tekscan was compared to that of the JAMAR digital dynamometer by performing a linear regression and calculating Pearson’s R. To assess the consistency of the Tekscan within a study visit, the slope of participants’ MVC forces for each hand were calculated. The slopes were plotted in a histogram, and a one-sample *t*-test was performed with a comparator of zero.

#### 2.3.2. Isometric Pinch Individuation

To quantify each participant’s ability to perform isometric pinch grip [1,2,29,34], isometric individuation scores (IIS) were calculated using Equation (1), as shown below [1,2,29,34]. Here, the force of the indicated finger is divided by the sum of the force applied by all four fingers, essentially calculating a percentage of the total force applied to the mat by the digit of interest. The thumb and palm of the hand were excluded. Scores were calculated on a scale of zero to one with one being a theoretically “ideal” individuation (100%); zero is the “poorest” individuation (0%).

**Figure 3:**
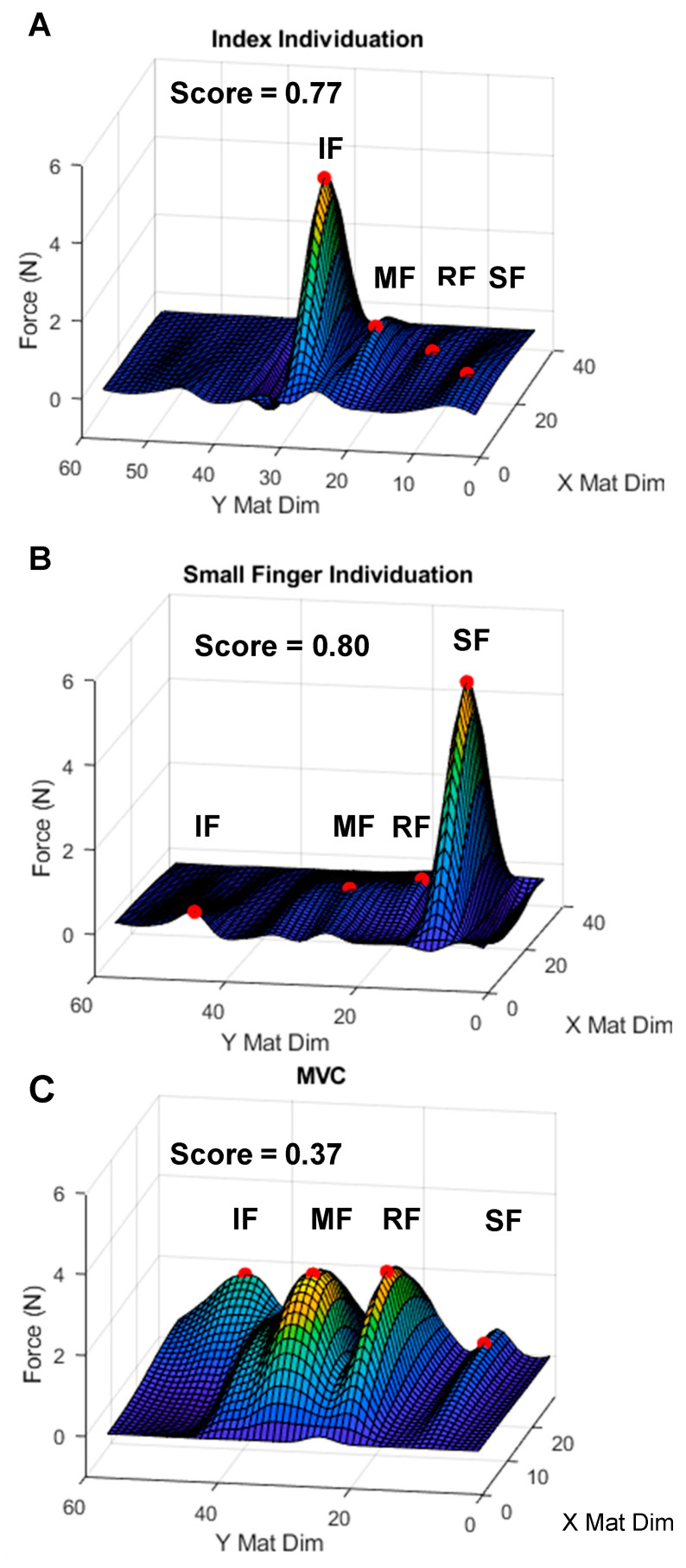
Representative cubic interpolated maps of hand forces during isometric pinch and whole-hand grasp tasks. Heatmap of forces applied to the Tekscan F-socket at the time of maximum total force during an individual task. The Y Mat Dim is perpendicular to the orientation of the four non-thumb fingers. Red points indicate the four greatest peaks on the heatmap used in calculating individuation scores. Individuation scores are displayed. (**A**) Index finger individuation. (**B**) Small finger individuation. (**C**) Whole-hand grasp. Mat Dim = pressure mat dimension. IF = index finger, MF = middle finger, RF = ring finger, and SF = small finger.

For each pinch task, the timepoint in which the greatest total force recorded via the Tekscan was identified and used to calculate the IIS. A cubic interpolated map of data recorded at this time was developed using the interp2 MATLAB function [53]. From there, the extrema2 MATLAB function was used to identify the four greatest peaks in the interpolated map as representations of the four fingers engaged in the task of isometric individuation [54]. The greatest peak identified served as a proxy of force applied by the indicated finger (*F_i_*), while the sum of all four peaks were represented by the forces applied by all four fingers (*F_all_*). In rare instances, it was not possible to identify one or more of the three peaks to represent the non-indicated fingers because engagement of the non-indicated digits was so limited. In these few cases, the mean of the interpolated map in areas of the non-indicated digits was used as a proxy for one of the three non-indicated digits (Figure 3).

*Isometric Individuation Score (IIS)*

(1) $$\mathrm{Score}=\frac{F_{i}}{\sum F_{all}}$$

To visualize the distributions of IISs, violin and Bland–Altman plots were developed using the violin and Bland–Altman functions, respectively [55,56]. Repeatability of the IIS metric was assessed via the ICCs calculated by correlating the participants’ mean individuation scores for a given task in the first visit to that of the second visit [52]. MDC was calculated using methods described above [57]. Wilcoxon signed rank tests compared scores from the first visit to the second. Finally, IIS were calculated using data from whole-hand MVC trials (i.e., theoretically poor individuation trials) to further understand their practical range [1,2,29,34,40].

## 3. Results

### 3.1. Whole-Hand Grasp Strength

**Figure 4:**
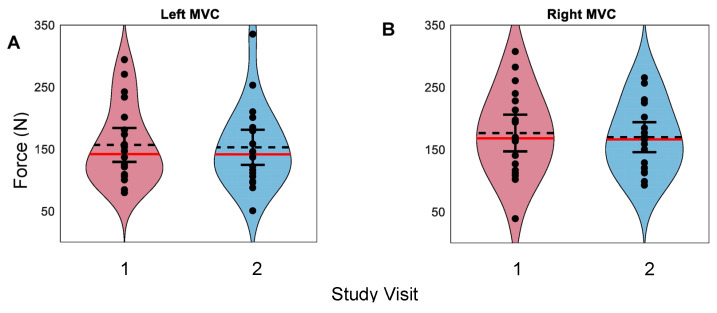
MVC across participants and visits. Left (**A**) and right (**B**) hands. Participants represented by points. Dashed lines indicate sample means. Red lines indicate sample medians. Error bars represent 95% confidence intervals.

**Figure 5:**
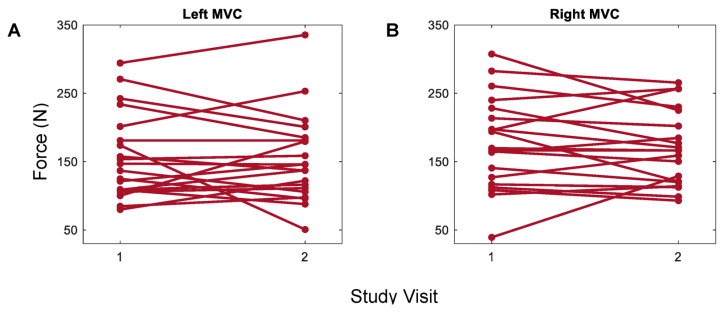
Within-participant change in MVC across visits. Left (**A**) and right (**B**) hands. Minimal changes were observed within participants between visits.

**Figure 6:**
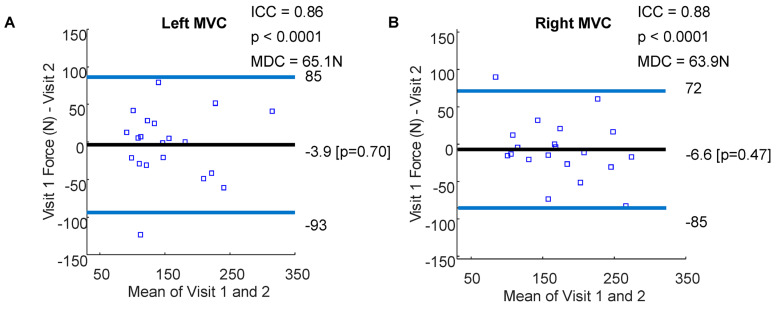
Repeatability MVC. Bland–Altman plots of individuation scores for the left (**A**) and right (**B**) hands. The horizontal black lines represent the mean change in force between the two visits with the blue horizontal lines representing 1.96 standard deviations above and below the means. Participant-level data are represented with the blue squares. ICCs, corresponding *p*-values, and minimal detectable changes are shown.

For trials of grasp strength, mean forces fell between 40 N and 350 N for both left and right hands. Overlapping 95% confidence intervals between the first and second visits for both the left and right hands were observed (Figure 4). Some changes in MVC forces measured via Tekscan from visit 1 to visit 2 were observed for both the left and right hands within the participants (Figure 5). MVC ICCs were 0.86 (*p* < 0.0001) and 0.88 (*p* < 0.0001) for the left and right hands, respectively, suggesting ‘good’ repeatability (Figure 6) [58,59]. The MDC’s were 65.1 N and 63.9 N for the left and right hands, respectively. ICCs of MVC obtained via the JAMAR dynamometer were 0.96 and 0.97 for the left and right hands, respectively, which is consistent with prior validity studies [24]. MDCs of JAMAR data were 68 N and 58 N for the left and right hands, respectively [24].

**Figure 7:**
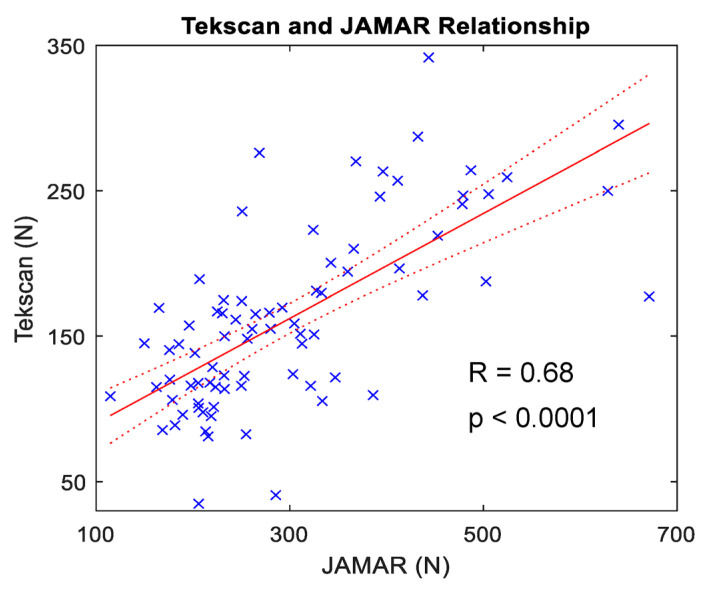
Correlation between MVC measurements from the Tekscan pressure grid and JAMAR dynamometer. Linear regression comparing the first MVC trial with the Tekscan F-Socket with that of the JAMAR digital dynamometer. Pearson’s R is shown. The solid red line represents linear regression line of best fit, and dashed red lines indicate the 95% confidence interval. Participant-level data indicated by the blue X’s. Force data from Tekscan and JAMAR show a moderately positive correlation.

**Figure 8:**
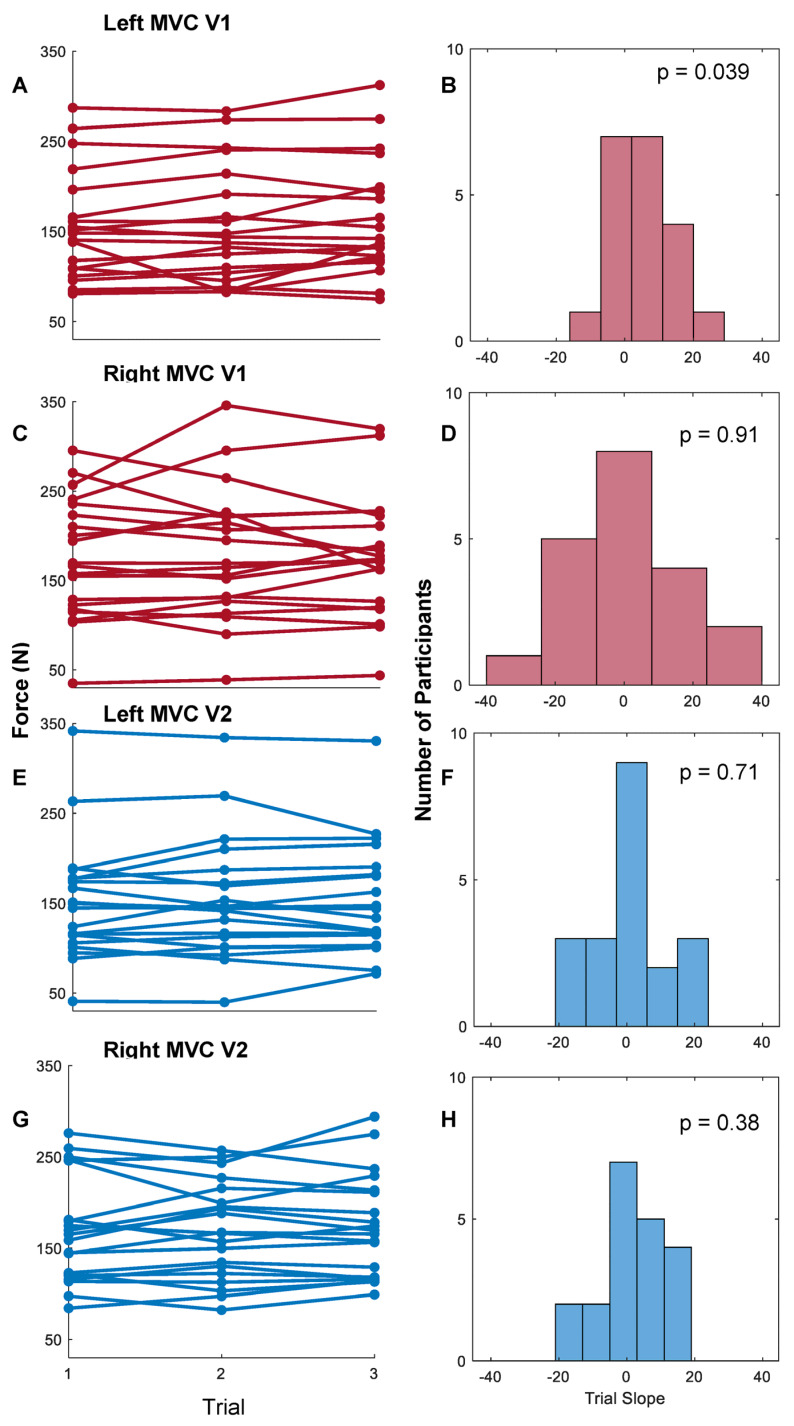
Within-participant and within-visit MVC changes across attempts. (**A**,**C**,**E**,**G**) Connected points represent participants’ trial forces. (**B**,**D**,**F**,**H**) The slope of participants’ trials were calculated and are shown in a histogram. One-sample *t*-tests with a comparator of zero were performed, and *p*-values are shown. MVC was grossly stable between trials for a given visit and hand.

When the first full-effort trials with the Tekscan F-Socket were correlated with that of the JAMAR digital dynamometer, Pearson’s R was 0.68 (*p* < 0.0001), demonstrating a moderately strong positive correlation (Figure 7). Minimal change was observed between trials of whole-hand grasp for both the left and right hands at visits one and two (Figure 8A,C,E,G). To determine the stability of whole-hand grasp rials during a given visit, the slope of participants individuation scores in each study visits were calculated (Figure 8B,D,F,H).

One-sample *t*-tests between participants’ slopes and a comparator of zero revealed a lack of significant difference for the right hand at both the first (*p* = 0.91) and second (*p* = 0.38) visits (Figure 8D,H). A similar lack of significant difference was observed for the left hand in the second visit (*p* = 0.71) (Figure 8F), but a weak significant positive skew was observed for participants’ left-hand trials at the first visit (*p* = 0.039) (Figure 8B). Taken together, the lack of significant difference from zero suggests that the flexible pressure grid consistently recorded MVC forces for the right hand during both study visits. However, for the left hand, there was an increase in MVC forces during trials from the first visit and stable recordings from the second visit.

### 3.2. Isometric Pinch Individuation

**Figure 9:**
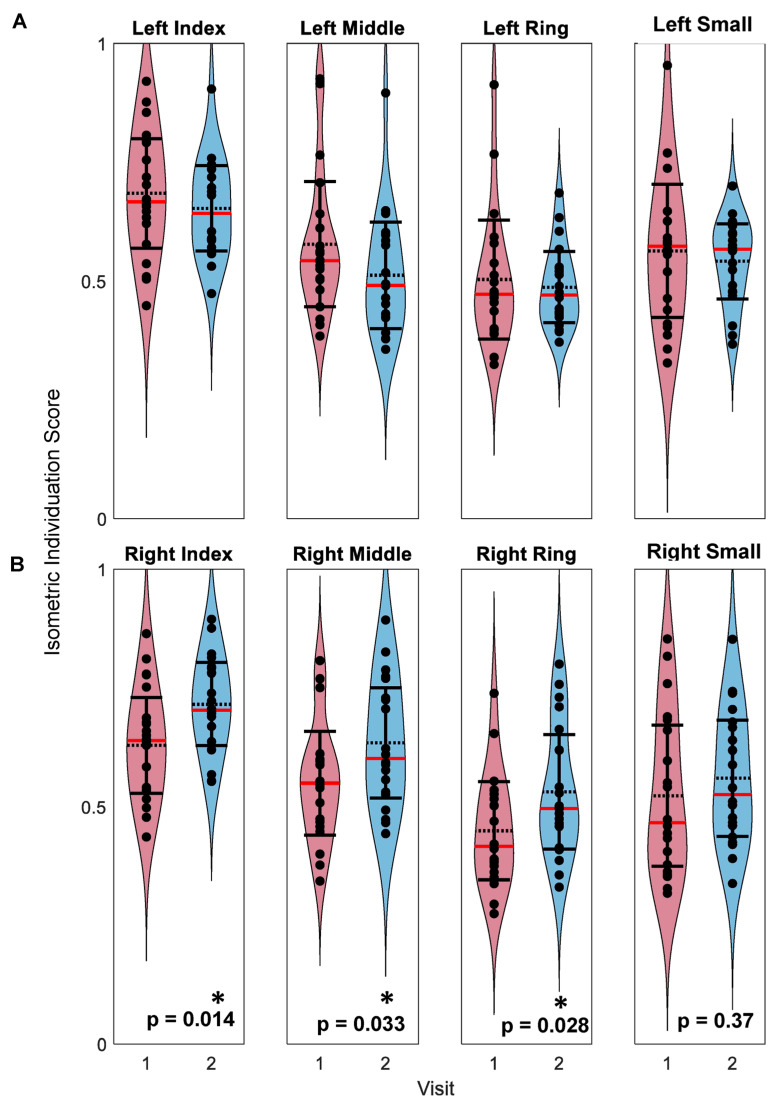
Distribution of isometric individuation scores. Points represent participants’ mean individuation scores for left (**A**) and right (**B**) hands with distributions from the first visit shown in red and from the second visit shown in blue. Horizontal, black, dashed lines represent the sample means. Horizontal red lines represent the sample medians. Error bars indicate 95% confidence intervals. Paired *t*-tests were performed to compare first and second visit scores, and *p*-values are shown with significant differences indicated by asterisks (*p* < 0.05).

**Figure 10:**
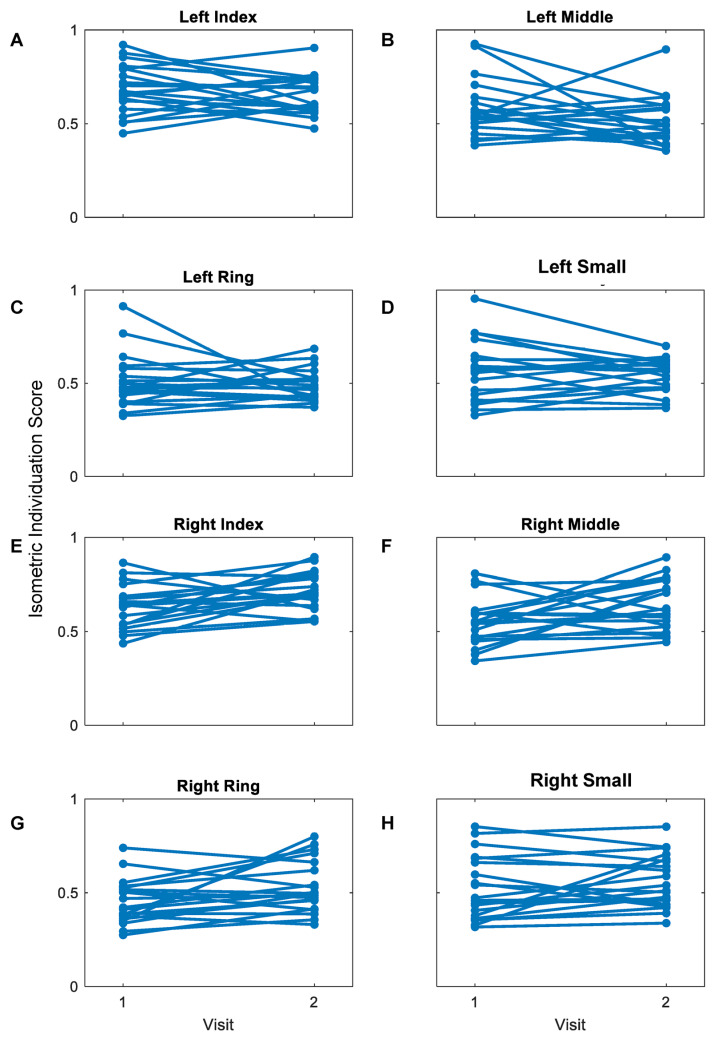
Within-participant changes in mean isometric individuation scores between two visits. There was minimal change in participants’ mean kinetic individuation scores between the two visits for all fingers of the left hand (**A**–**D**). An increase in kinetic individuation scores is observed for the right index, middle, and ring fingers for the second visit compared to the first (**E**–**G**). There is minimal change observed for the right small finger scores (**H**).

Mean IISs for both the left and right hands were widely distributed between 0.30 and 1.0 (Figure 9). Wilcoxon signed rank tests revealed non-significant differences between the first and second visits for the left index, middle, ring and small fingers (*p* = 0.35, *p* = 0.14, *p* = 0.88, and *p* = 0.55, respectively) (Figure 9A) as well as the right small finger (*p* = 0.37) (Figure 9B). For the right index, middle, and ring fingers, isometric individuation scores significantly increased from the first to the second visits (*p* = 0.014, *p* = 0.033, and *p* = 0.028, respectively) (Figure 9B). Minimal change was observed within participants’ left hand individuation scores (Figure 10A–D). A general increase in right index, middle, and ring finger individuation scores was observed (Figure 10E–G), but minimal change was observed within participants’ right small finger scores (Figure 10F–H).

**Figure 11:**
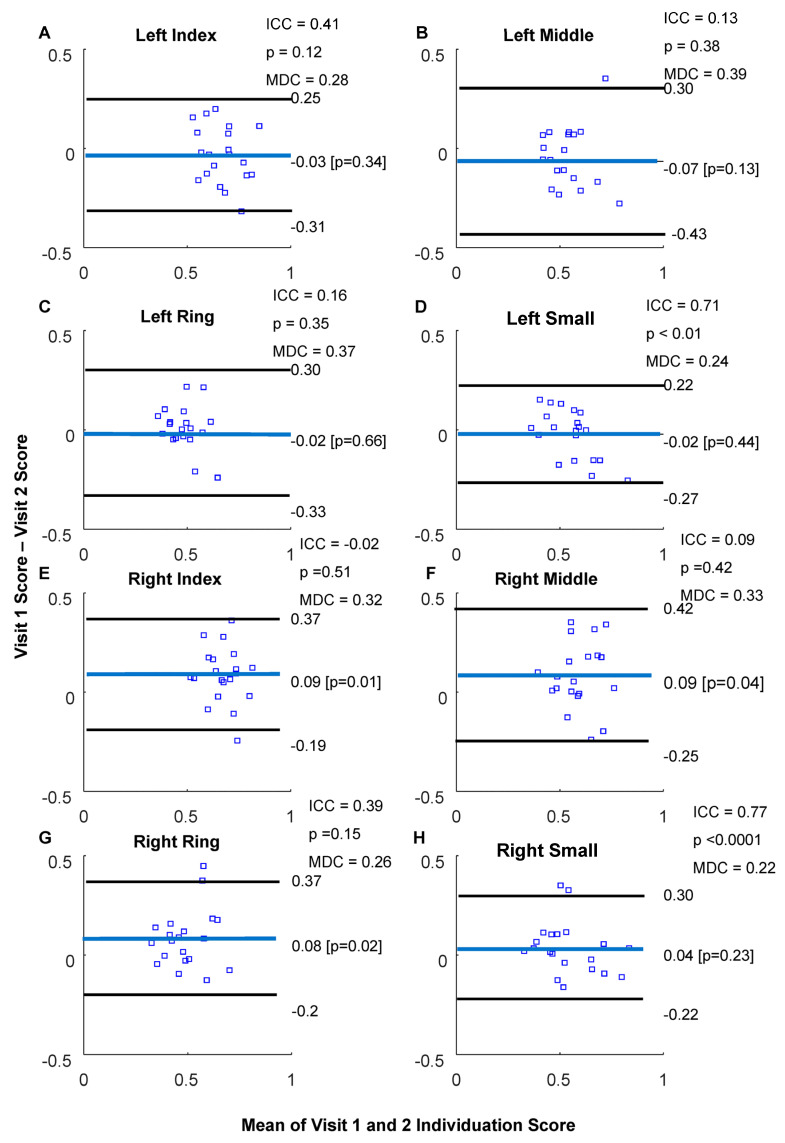
Bland–Altman plots of kinetic individuation scores. (**A**–**D**) Left BA plots with ICC, MDC, and *p*-values. (**E**–**H**) for right ICC = intra-class correlation coefficient. MDC = minimal detectable change. Blue squares represent participant means, Horizontal black and bluelines indicate mean difference and +/−1.96 standard deviations, respectively.

Left-hand ICCs were 0.41 (*p* = 0.12), 0.13 (*p* = 0.38), 0.16 (*p* = 0.35), and 0.71 (*p* < 0.01) for the index, middle, ring, and small fingers, respectively. Left-hand MDCs were 0.28, 0.39, 0.37, and 0.24 for the index, middle, ring, and small fingers, respectively (Figure 11A–D). Right-hand ICCs were −0.02 (*p* = 0.51), 0.09 (*p* = 0.42), 0.39 (*p* = 0.15), and 0.77 (*p* < 0.0001) for the index, middle, ring, and small fingers, respectively. Right-hand MDCs were 0.32, 0.33, 0.26, and 0.22 for the index, middle, ring, and small fingers, respectively (Figure 11E–H).

**Figure 12:**
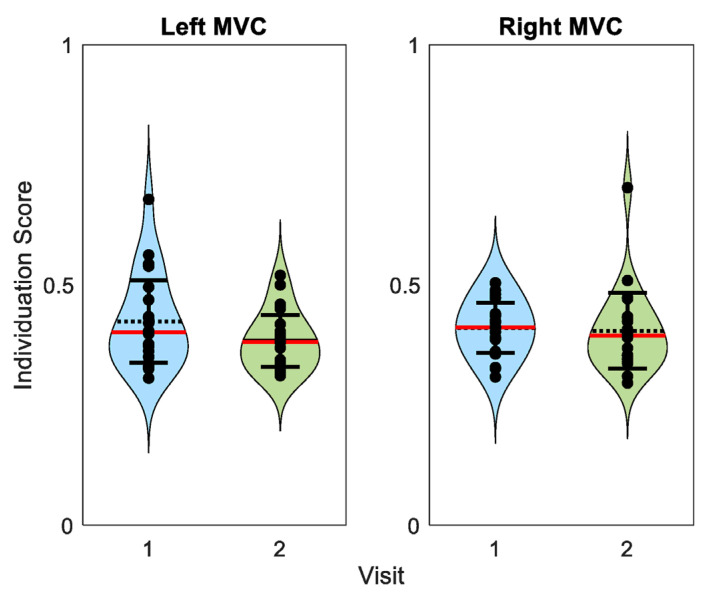
Individuation scores calculated from whole-hand grasp trials. Dashed black lines represent sample means. Red lines represent sample medians. Error bars represent 95% confidence intervals. Points represent participant means. Scores were expectedly low and ranging from 0.30 to 0.70.

To assess the possible range of individuation scores, we used MVC trials (which should represent very poor individuation performance) to calculate the isometric individuation scores on the same scale. Both left and right MVC trials from the first and second visits produced scores ranging from 0.30 to 0.70 with overlapping 95% confidence intervals (Figure 12).

## 4. Discussion

The purpose of this study was to assess whether quantitative measurements of whole-hand strength and isometric pinch individuation obtained using a commercially available flexible pressure sensor grid might address a critical need in multiple surgical and rehabilitative specialties: an objective and repeatable assessment platform consistent across time and location [1,2]. Although the Tekscan F-socket has been used in a variety of clinical and research settings [60,61,62], to our knowledge, we are the first group to test it for this purpose. While we found MVC measurements to be repeatable and well-correlated to a validated digital dynamometer, measurements of isometric pinch individuation were not sufficiently repeatable across visits to be a clinically useful tool. Potential reasons for the latter finding are expounded below.

### 4.1. Maximum Voluntary Contraction Using a Flexible Pressure Grid

In our cohort of healthy volunteers, we noted good repeatability of MVC measurement in each hand across two visits [41,42]. Despite good ICCs, some participants’ mean forces did vary individually from the first to the second visit. Explanations for observed force variances may include greater familiarity with the task resulting in the achievement of higher forces at visit two, or subjects’ fingers may have changed positions on the Tekscan, as there was limited sensor density leaving blank spaces that may have been squeezed without detecting the force. MDCs indicated that a change of applying ~64 N, approximately equivalent to the weight of a gallon of paint, would be associated with a noticeable change using this device. However, MDCs calculated with data collected from the JAMAR in this study were also ~63 N, suggesting similar performance of the JAMAR and Tekscan pressure grid in this regard.

**Figure 13:**
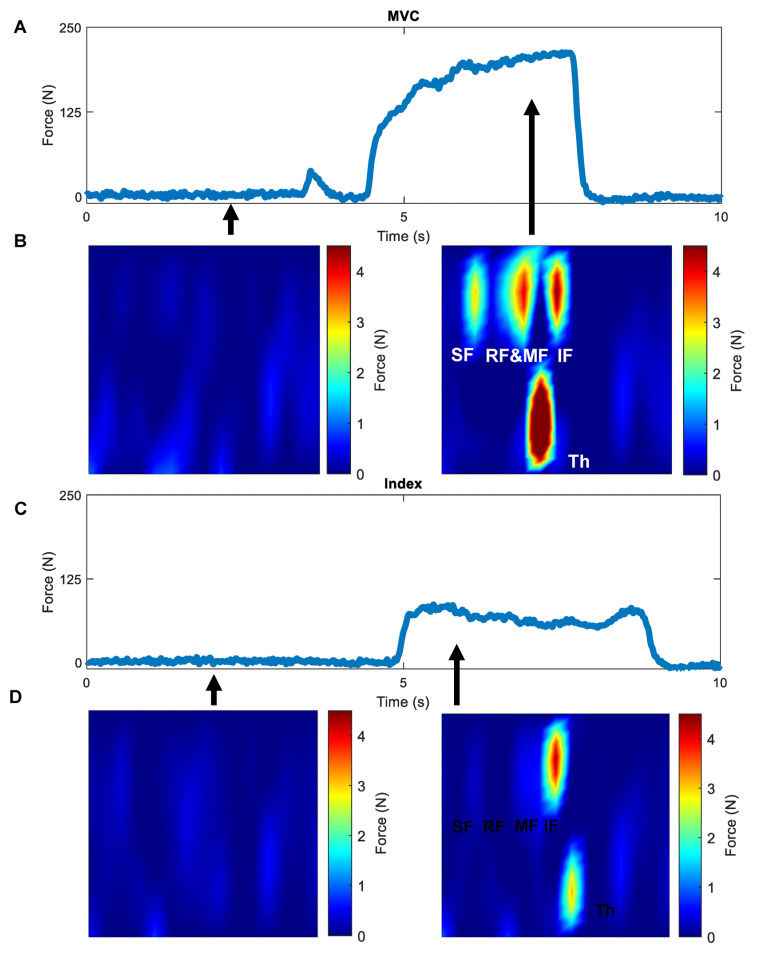
Constructing heatmaps from Tekscan data. Force data from trials of whole-hand maximum voluntary contraction (MVC) (**A**) and index isometric pinch grip (**C**) can be used to construct heatmaps (**B**,**D**). (Th = thumb, IF = index finger, MF = middle finger, RF = ring finger, and SF = small finger).

The positive correlation between MVC measurements obtained with the Tekscan F-socket and JAMAR dynamometer suggests the Tekscan may be useful for this purpose. One potential advantage of the Tekscan F-Socket in this setting is its ability to construct a heatmap (i.e., distribution) of hand forces, as well as its ability to track forces over time during a given trial (Figure 13), whereas the JAMAR digital dynamometer provides only a single value of maximal force applied to the device [24,63]. While the use of such a heat map is intriguing, data from this study cautions that the repeatability and validity of such a real-time heat map needs to be directly demonstrated prior to clinical application.

### 4.2. Isometric Individuation

Isometric individuation scores calculate the percentage of force applied by the indicated finger out of the total force applied by all four non-thumb digits. Ideally, higher scores imply less co-contraction. Between the left and right hands and for the first and second visits, the index fingers achieved the highest individuation scores, whereas the ring and little fingers typically scored the lowest. The higher scores reached by the index finger were expected, given there are multiple muscles acting independently on the index finger, thereby enhancing its relative dexterity [64]. There were significant increases in right index, middle, and ring individuation scores from the first to the second visit, while all the other fingers demonstrated non-significant differences. The differences could at least partially be due to greater familiarity with the task at the second visit contributing to enhanced performance as well as variations in finger placement on the force sensors between the two visits. However, the ICCs for nearly all fingers were weak, indicating poor repeatability of these measurements. The left small finger showed moderate repeatability and the right small finger showed good repeatability. Such a discrepancy as well as the general poor repeatability of other fingers indicate this method of assessing isometric individuation shows it is not likely translatable in its current form. Unfortunately, there is still no standardized, validated, repeatable way to assess isometric individuation [1,2,33,36,42,65].

Findings presented herein can inform future studies aimed at improving repeatability of the IIS metric either by standardizing finger placement in a more rigorous manner or by developing novel devices for this purpose. While other techniques have been explored, such as using separate load cells for each finger [1,2], there remains limited assessments of repeatability of these techniques [2,35,36]. Moreover, though a load cell design has certain advantages, a flexible pressure grid can uniquely indicate specific areas within the hand and fingers that may not be applying the expected force, potentially adding to diagnostic and rehabilitative applications. Improving the methods for measuring isometric pinch individuation will be the focus of our future work.

### 4.3. Clinical and Research Applications

The authors of this manuscript have diverse backgrounds with a common interest in hand therapeutics related to central or peripheral nervous system injury [9,12,66,67,68,69]. Although many groups have studied hand strength and function, there is no standardized, objective, and repeatable platform to assess isometric pinch individuation that can be shared across specialties and facilities over time [70,71,72,73,74]. There is a real need to quantitatively track clinical outcomes in pre-, intra-, and post-operative settings, as well as longitudinally across natural histories of disease and throughout rehabilitative interventions. Furthermore, the development of novel treatments and the ability to compare their efficacy to current interventions will rely on accurate and repeatable metrics. Here, we attempted to bridge this gap using a commercially available flexible pressure sensor grid that is easily accessible and broadly applicable. Unfortunately, as it stands, measurements of isometric pinch individuation with this device were not sufficiently repeatable to be clinically meaningful. Therefore, future endeavors will focus on improving measurement protocols and potentially developing a novel device designed specifically to obtain measurements of isometric pinch individuation [74].

### 4.4. Limitations

Our sample of healthy volunteers consisted primarily of young adult right-handed females from a single region within the midwestern United States. Therefore, extrapolations to other demographic populations should be made with caution or not at all. However, because this was a study of device measurement repeatability and not an intervention, it was important to use a homogenous study group such as this to avoid potential confounders [5,18].

## 5. Conclusions

In this study, we assessed the repeatability of isometric pinch individuation and MVC measurements using the commercially available Tekscan F-Socket flexible pressure sensor grid. While we showed acceptable repeatability for MVC measurements, isometric pinch grip assessments were not consistent within subjects across visits. Potential reasons for this include insufficient grid density and/or inconsistent hand/finger placement. Future endeavors may include custom designs to address these issues. We hope these data will inform the continued development of shareable devices that will be able to sufficiently quantify metrics of hand strength and function across varied clinical and research platforms. Specifically, we believe these results will inform future research as well as diagnostic and rehabilitative paradigms for a variety of medical specialties interacting with hand strength and function, such as neuro-, orthopedic, and plastic surgery, as well as physical medicine and rehabilitation and physical/occupational therapy.

## Figures and Tables

**Table 1:** Participant demographics.

Participant Demographics	Characteristics	Number of Participants (Percentage)
Sex	Male	16 (80%)
	Female	4 (20%)
Handedness	Right	20 (100%)
	Left	0 (0%)
Age	Mean ± Standard Deviation	28.8 ± 2.5
	Median	27.5
	Minimum	23
	Maximum	55

## Data Availability

All necessary data supporting these results are available in the manuscript figures and text. If readers have any further questions regarding the data and results, they can contact the corresponding author.
